# Key Considerations for Incorporating Conversational AI in Psychotherapy

**DOI:** 10.3389/fpsyt.2019.00746

**Published:** 2019-10-18

**Authors:** Adam S. Miner, Nigam Shah, Kim D. Bullock, Bruce A. Arnow, Jeremy Bailenson, Jeff Hancock

**Affiliations:** ^1^Department of Psychiatry and Behavioral Sciences, Stanford University School of Medicine, Stanford, CA, United States; ^2^Department of Epidemiology and Population Health, Stanford University School of Medicine, Stanford, CA, United States; ^3^Department of Communication, Stanford University, Stanford, CA, United States; ^4^Stanford Center for Biomedical Informatics Research, Stanford University School of Medicine, Stanford, CA, United States

**Keywords:** natural language processing, artificial intelligence, expert systems, psychotherapy, conversational AI, chatbot, digital assistant, human–computer interaction

## Abstract

Conversational artificial intelligence (AI) is changing the way mental health care is delivered. By gathering diagnostic information, facilitating treatment, and reviewing clinician behavior, conversational AI is poised to impact traditional approaches to delivering psychotherapy. While this transition is not disconnected from existing professional services, specific formulations of clinician-AI collaboration and migration paths between forms remain vague. In this viewpoint, we introduce four approaches to AI-human integration in mental health service delivery. To inform future research and policy, these four approaches are addressed through four dimensions of impact: access to care, quality, clinician-patient relationship, and patient self-disclosure and sharing. Although many research questions are yet to be investigated, we view safety, trust, and oversight as crucial first steps. If conversational AI isn’t safe it should not be used, and if it isn’t trusted, it won’t be. In order to assess safety, trust, interfaces, procedures, and system level workflows, oversight and collaboration is needed between AI systems, patients, clinicians, and administrators.

## Introduction

Clinicians engage in conversations with patients to establish a patient-therapist relationship (i.e., alliance), make diagnoses, and provide treatment. In traditional psychotherapy, this conversation typically involves a single patient and a single clinician (1). This model of psychotherapy is being modified because software programs that talk like people (i.e., conversational artificial intelligence, chatbots, digital assistants) are now beginning to provide mental health care (2). Conversational artificial intelligence (AI) is gathering diagnostic information (3, 4) and delivering evidence-based psychological interventions (5–7). Additionally, conversational AI is providing clinicians with feedback on their psychotherapy (8) and talking to young people about suicide, sex, and drug use (9, 10).

Conversational AI appears unlikely to achieve enough technical sophistication to replace human therapists anytime soon. However, it does not need to pass the Turing Test (i.e., able to hold human seeming conversations) to have a significant impact on mental health care (2). A more proximal challenge is to plan and execute collaborative tasks between relatively simple AI systems and human practitioners (11–13). Although AI in mental health has been discussed broadly (for a review see 14), specific formulations of clinician-AI collaboration and migration paths between forms remain vague.

Articulating different forms of collaboration is important, because the deployment of conversational AI into mental health diagnosis and treatment will be embedded within existing professional services. Conversational AI will likely interact with traditional workers (i.e., clinicians), but how these roles and responsibilities will be allocated between them has not been defined. To guide future research, we outline four approaches and dimensions of care that AI will affect.

Within the four approaches of AI-human integration in mental health service delivery, one extreme is a view that any involvement by conversational AI is unreasonable, putting both patients and providers at risk of harmful unintended consequences. At the other extreme, we explore how conversational AI might uniquely serve a patient’s needs and surpass the capacity of even the most experienced and caring clinician by overcoming entrenched barriers to access. Although embodiment (e.g., virtual avatars or robots) can have a significant impact on interactions with virtual systems, we focus exclusively on the potential benefits and challenges of verbal and written language-based conversation and ignore the implications of embodiment or presence (15). **Table 1** summarizes the four approaches and our related assumptions.

**Table 1:** Delivery approaches and dimensions of impact for conversational AI.

Care delivery approach	Dimensions of impact			
	Access to care	Quality	Clinician-patient relationship	Patient self-disclosure and sharing
**Humans only**	Unchanged	Established	No disruption	Unchanged
**Human delivered, AI informed**	Unchanged	Potentially improved	Potentially disrupted^a^	Unknown
**AI delivered, human supervised**	Improved, but limited scalability	Unknown	Likely disrupted	Unknown
**AI only**	Improved, not restrained by human attention	Unknown	Nonexistent	Unknown

a By “disrupt” we do not mean to signal that the result will be necessarily good or bad.

## Care Delivery Approaches

It is unclear whether the path forward will involve simultaneous experimentation with all four degrees of digitization, or progression through these approaches. We first briefly describe how these compare to the way individual psychotherapy is most often delivered today. Perhaps surprisingly, laws, norms and the ethics of data sharing represent a nonobvious but critical factor in how these alternative approaches can operate now or develop in the future.

Currently, psychotherapy sessions are rarely recorded except in training institutions for supervision. When they are, for example during training or to assess clinician fidelity during clinical trials, trained human clinicians with prescribed roles and responsibilities are the listeners and provide oversight. With few exceptions, such as immediate risk of serious harm to the patient or others, clinicians need explicit permission to share identifiable patient information. When one of these exceptions is invoked, there is an obligation to limit the sharing strictly to the extent needed to provide effective treatment and ensure safety (16, 17). Against this backdrop, having conversational AI listen to psychotherapy sessions or talk directly with patients represents a departure from established practice.

In the “humans only” approach, psychotherapy remains unchanged. Most psychotherapy sessions are heard only by the patient and clinician who are in the room. If a session were recorded, the labor intensiveness of human review would ensure most sessions would never be analyzed (8). The second approach, “human delivered, AI informed,” introduces into the room a listening device connected to software that detects clinically relevant information (18) such as symptoms or interventions (19), and relays this information back to the patient or clinician. Quantitative analysis of recorded psychotherapy is in its early stages, but it shifts to software programs the burden of extracting relevant information from audio or text. In the third approach, “AI delivered, human supervised,” patients speak directly to a conversational AI with the goal of establishing diagnoses or providing treatment (20). A human clinician would either screen patients and hand off specific tasks to conversational AI or supervise conversations between front-line conversational AI and patients. The fourth approach, “AI only,” would have patients talk to a conversational AI with no expectation of supervision by a human clinician.

One of the less developed but more alluring ideas of AI psychotherapy is “AI delivered, human supervised.” Even the most ardent supporters of AI will acknowledge that there are certain things humans do better than computers. Combining people and algorithms may potentially build on the best of both approaches, and AI–human collaboration has been suggested as a way to address limitations in planning treatment in other medical areas such as oncology (21). Indeed, the prevailing opinion of expert systems researchers in the 1980s argued that computer–human collaboration would outperform either people or computers alone (for a review see 22).

In assessing any system to augment the practice of psychotherapy the first consideration of its impact should be that it will ensure patients and clinicians are helped and not harmed (23, 24). In the discussion below, we consider salient issues that impact the potential value and harm of different delivery mechanisms by focusing on four dimensions of impact: access to care, quality, clinician-patient relationship, and patient self-disclosure.

## Dimensions of Impact

### Access to Care

Limited access to mental health treatment creates a demand for scalable and non-consumable interventions (25, 26). Despite the high costs and disease burden associated with mental illness (27), we have a decreasing number of clinicians per capita available to provide treatment in the US (28). Increasing the number of human clinicians is not currently feasible, in part because of the decline from 2008 to 2013 per capita for both psychologists (from a ratio of 1:3,642 to 1:3,802) and psychiatrists (from a ratio of 1:7,825 to 1:8,476) (28). Conversational AI has the potential to help address insufficient clinician availability because it is not inherently limited by human clinician time or attention. Conversational AI could also bridge one of the current tensions in care delivery: although clinicians value patient conversations, they have no financial incentive to engage in meaningful but lengthy conversations (29).

The decreasing amount of time spent in meaningful conversations exacerbates the shortage of psychiatrists and psychologists. Psychiatrists’ use of talk therapy has been consistently and steadily declining, meaning fewer patients are receiving talk therapy during psychiatric visits (30). In contrast to a human clinician’s time and attention, conversational AI is relatively non-consumable, making it an attractive alternative to delivery of care by a human. If conversational AI is effective and acceptable to both patients and clinicians, it may address longstanding challenges to mental health access. These include the ability to accommodate rural populations and to facilitate increased engagement from people who may experience traditional talk therapy as stigmatizing (31).

### Quality

Technology has been highlighted as a way to better understand and disseminate high quality psychotherapy (32, 33). Clinicians are already using texting services to deliver mental health interventions (34), which demonstrates a willingness by patients and clinicians to test new approaches to patient-clinician interaction. These new approaches facilitate novel measures of intervention quality. For example, innovations in computer science (e.g., natural language processing and machine learning) are being used to assess language patterns of successful crisis interventions in text-based mental health conversations (18, 35). Computational analysis of psychotherapy is encouraging researchers and companies to identify patterns of patient symptomology and therapist intervention (36, 37). This approach may improve psychotherapy quality by better understanding what effective clinicians actually do. This assessment has historically occurred through clinicians’ self-reports or time intensive human audits (e.g., 38).

Although its efficacy is not definitively established, there are reasons to expect that conversational AI could constructively enhance mental health diagnosis and treatment delivery (39, 40). A diagnostic interview aids the patient and clinician in understanding the patient’s presenting problem and provides a working model of how problems are being maintained. Approaches vary from highly structured diagnostic interviews [e.g., Structured Clinical Interview for DSM-5 (41)] to unstructured interviews in which the conversation develops based on the clinician’s expertise, training, and the patient’s features. Conversational AIs have interviewed patients about symptoms for PTSD with a high level of patient acceptance (20). Conversational AI has been piloted across numerous clinically relevant groups such as clinical depression (6) and adolescent stress (42). In a study in which students believed they were speaking with a conversational AI, the students reported feeling better after talking about their problems following the encounter (43). Although these early findings point to potential benefits, there is a lack of rigorous clinical trial data and uncertainty about regulatory oversight (2).

Yet while there is reason for optimism, inflated or unsubstantiated expectations may frustrate patients and weaken their trust in psychotherapeutic interventions (44, 45). Many current computation methods can be used to search for specific dialogue acts, but additional work is needed to map theoretically important constructs (e.g., therapeutic alliance) to causal relationships between language patterns and clinically relevant outcomes. Psychotherapy quality will be difficult to assess without disentangling causal inferences and confounding factors. Beyond computation, patients’ attitudes matter in psychotherapy because those who have a negative experience compared with their expectations have worse clinical outcomes (46). If a patient loses trust in a conversational AI, they may be less likely to trust human clinicians as well. As conversational AI becomes more sophisticated and expectations of benefit increase, there are growing concerns that users will transition from feeling let down to feeling betrayed (47). These factors suggest that careful experimentation about sub-processes in AI-mediated communication merits research attention.

### Clinician–Patient Relationship

Modern medicine views the patient–clinician relationship as critical to patient health (48), and provider wellness (49). Indeed, appreciation of the importance of the patient–clinician relationship in modern medicine can be traced back to the influence of clinical psychology (50). Therapeutic alliance develops from clinicians’ collaborative engagement with patients and reflects agreement on treatment goals, the tasks necessary to achieve such goals, and the affective bond between patient and provider (51). Therapeutic alliance is consistently associated with symptom improvement in psychotherapy (52–54). Numerous approaches exist to create alliance during psychotherapy, including the use of supportive language, mirroring emotions, and projecting warmth. Although originally conceptualized for human-to-human conversations, users have reported experiencing a sense of therapeutic alliance when speaking directly with conversational AI, suggesting this bond may not necessarily be restricted to human-human relationships (3). If conversational AI can create and maintain a therapeutic alliance, the provision of psychotherapy will not be necessarily limited by human clinicians’ time and attention.

Establishing therapeutic alliance with conversational AI may benefit both patients and providers. By allowing conversational AI to take over repetitive, time-consuming tasks, clinicians’ attention and skill could be deployed more judiciously (55). Allowing clinicians to do less of the work that contributes to burnout, such as repetitive tasks performed with little autonomy, may improve clinicians’ job satisfaction (56). Clinician burnout is associated with worse patient outcomes and is increasingly recognized as a problem which must be more adequately addressed (57, 58).

At the same time, software that augments clinical duties has been criticized for distancing clinicians from patient care (59). In mental health, this risk is especially salient because the content of therapy is often quite intimate. Some of the repetitive, time-consuming tasks clinicians engage in with patients, such as reviewing symptoms or taking their history, are precisely the vehicles by which clinicians connect with and understand their patients’ experiences and develop rapport. It is unknown whether having a conversational AI listen in on psychotherapy will significantly impact patients’ and clinicians’ sense of therapeutic alliance. This area merits further research.

### Patient Self-Disclosure and Sharing

Patient self-disclosure of personal information is crucial for successful therapy, including sensitive topics such as trauma, substance use, sexual history, forensic history, and thoughts of self-harm. Patient self-disclosures during psychotherapy are legally and ethically protected (24) and professional norms and laws have been established to set boundaries for what a clinician can share (60). Unauthorized sharing of identifiable patient information can result in fines, loss of license, or even incarceration. Moreover, because of the natural limitations of human memory, patients are unlikely to expect a human clinician to remember entire conversations perfectly in perpetuity. This capacity is in stark contrast to conversational AI, which has near-limitless capacity to hear, remember, share, and analyze conversations as long as desired. Because humans and machines have such different capacities, patient expectations of AI capabilities may impact treatment decisions and consent to data sharing (23).

In mental health, conversational AI has been shown to both facilitate and impede disclosure in different contexts. For example, users were more open with a conversational AI than with a human listener in reporting mental health symptoms (20), and have been successfully used to treat persecutory delusions for people with psychosis (61). Conversely, users were more reluctant to disclose sensitive information such as binge drinking behavior to a conversational AI compared to a non-responsive questionnaire (62). Because personal disclosures are central to diagnosis and treatment in psychotherapy, users’ expectations and behavior towards technology-mediated conversations merit further assessment (63, 64, 65).

Certain disclosures in a psychotherapy context carry specific ethical and legal mandates, such as reporting suicidal or homicidal ideation. In 1969, a therapist at the University of California did not share the homicidal ideation of a patient with the intended victim. The patient subsequently killed the named victim, and the victim’s family sued. This case (Tarasoff v. Regents of the University of California, 1974) established clinicians’ duty not only to protect the confidentiality of their patients but also to notify individuals their patient might harm. A failure to warn leaves a clinician liable to civil judgment (66). Most case law and norms have been established on the premise of a dyadic relationship between patient and clinician. The extent to which conversational AI inherits liability for harm is untested. As conversational AI takes on clinical duties and informs clinical judgment, expectations must be clarified about how and when these systems will respond to issues related to confidentiality, safety, and liability.

## Discussion

Experts in AI, clinicians, administrators, and other stakeholders recognize a need to more fully consider safety and trust in the design and deployment of new AI-based technologies (67, 68). A recent Lancet commission on global mental health states that “technology-based approaches might improve the reach of mental health services but could lose key human ingredients and, possibly, lower effectiveness of mental health care” (33). To inform future research directions, we have presented four approaches to integrating conversational AI into mental health delivery and discussed the dimensions of their impact.

Because conversational AI may augment the work of psychotherapy, we seek to encourage product designers, clinicians, and researchers to assess the impact of new practices on both patients and clinicians. Other areas of medicine have seen success with AI, such as lung cancer imaging and building diagnostic or prognostic models (69–73), and conversational AI for health is an emerging field with limited research on efficacy and safety (40, 63, 74).

Before we deploy AI-mediated treatment, workflow changes must be considered in the context of other demands on clinician time and training. Clinicians are already being asked to be familiar with telehealth (75) social media (76), and mobile health (77), while simultaneously being reminded of the need for self-care in light of clinician burnout (58). Before we insert new devices into clinical care, it will be crucial to engage clinicians and design evaluation strategies that appreciate the skills, attitudes, and knowledge of affected workers. Just as we can’t expect technology companies to easily understand healthcare, we can’t expect medical professionals to intuit or work in harmony with new technology without thoughtful design and training.

A limitation of this work is that we do not set out a specific research agenda, and some important considerations are beyond the scope of this work (e.g., the cost and feasibility of each approach). We propose instead that initiatives using conversational AI anticipate challenges and leverage lessons learned from existing approaches to deploying new technology in clinical settings that involve clinician training and patient protections from the start (32, 77). We instead encourage those proposing to put AI into care settings to directly consider and measure impact on access, quality, relationships, and data sharing.

The potential benefits are clear for mental health. If diagnosis or treatment can be done by conversational AI, the societal burden of treating mental health could be diminished. Additionally, conversational AI could have a more long-term relationship with a patient than clinicians who rotate out of training centers. Despite these potential benefits, technology carries risks related to privacy, bias, coercion, liability, and data sharing that could harm patients in expected (e.g., denial of health insurance) and unintended ways (33, 44, 74, 78, 79–81). Conversations are valuable for patients and clinicians, and it is crucial to make sure they are delivered safely and effectively, regardless of who or what does the talking.

## Author Contributions

ASM and JH contributed to the initial conceptualization and design of the manuscript. AM wrote the first draft. NS, KDB, BAA, and JB contributed to manuscript revision, read and approved the submitted version.

## Conflict of Interest

The authors declare that the research was conducted in the absence of any commercial or financial relationships that could be construed as a potential conflict of interest.
